# Mitochondrial DNA mutations in renal disease: an overview

**DOI:** 10.1007/s00467-019-04404-6

**Published:** 2020-01-10

**Authors:** Larissa P. Govers, Hakan R. Toka, Ali Hariri, Stephen B. Walsh, Detlef Bockenhauer

**Affiliations:** 1 Department of Renal Medicine, University College London, London, UK; 2 Manatee Kidney Diseases Consultants, Bradenton, USA; 3 Clinical Development, Sanofi Rare Disease, Boston, USA; 4 Renal Unit, Great Ormond Street Hospital for Children NHS Foundation Trust, Great Ormond Street, London, UK

**Keywords:** Mitochondrial DNA, Renal disease, Renal Fanconi syndrome, Distal tubulopathies, Nephrotic syndrome, Tubulointerstitial nephritis

## Abstract

Kidneys have a high energy demand to facilitate the reabsorption of the glomerular filtrate. For this reason, renal cells have a high density of mitochondria. Mitochondrial cytopathies can be the result of a mutation in both mitochondrial and nuclear DNA. Mitochondrial dysfunction can lead to a variety of renal manifestations. Examples of tubular manifestations are renal Fanconi Syndrome, which is often found in patients diagnosed with Kearns-Sayre and Pearson’s marrow-pancreas syndrome, and distal tubulopathies, which result in electrolyte disturbances such as hypomagnesemia. Nephrotic syndrome can be a glomerular manifestation of mitochondrial dysfunction and is typically associated with focal segmental glomerular sclerosis on histology. Tubulointerstitial nephritis can also be seen in mitochondrial cytopathies and may lead to end-stage renal disease. The underlying mechanisms of these cytopathies remain incompletely understood; therefore, current therapies focus mainly on symptom relief. A better understanding of the molecular disease mechanisms is critical in order to improve treatments.

## Introduction

Mitochondria are important organelles with the main function of converting the energy derived from oxidative phosphorylation into a “fuel” in the form of adenosine triphosphate (ATP), that can be used to catalyse cellular processes. Other important functions include calcium storage, regulation of metabolism and apoptosis, and cell signalling [1]. Mitochondria are present in all eukaryotic cells apart from mature red blood cells, which means that any organ has the possibility to be affected by mitochondrial dysfunction, resulting in a wide spectrum of manifestations [2]. Collectively, disorders of mitochondrial function are referred to as “mitochondrial cytopathies”. Even though our understanding of the mitochondria and its genome is increasing, the underlying mechanisms of these cytopathies remain incompletely understood to this day. Their estimated prevalence is around 1 in 5000, but this may be an underestimation as a substantial number of patients with a mitochondrial cytopathy caused by a mitochondrial DNA (mtDNA) mutation might be eluding diagnosis [3, 4].

Mitochondrial disorders are best known to affect the nervous system and muscles, but essentially all organs can be involved. The kidneys together with the heart have the highest energy demand of all organs when corrected for organ weight [5, 6]. Renal cells are therefore rich in mitochondria and depend on mitochondrial aerobic respiration to facilitate the energy-consuming task of reabsorption of the majority of the glomerular filtrate. Proximal tubulopathy is the most commonly recognised renal phenotype in children with mitochondrial disorders, since the proximal tubule not only has a high-energy demand but also lacks the capability to synthesis ATP anaerobically from glycolysis [7]. However, distal tubular defects, especially hypomagnesaemia, are also increasingly recognised as a renal manifestation of mitochondrial cytopathies [8]. The majority of mitochondrial proteins are encoded by the nuclear genome and mitochondrial cytopathies can therefore usually be explained by traditional Mendelian genetics. The mitochondrial genome and its genetics are, however, different from the nuclear genome and Mendelian genetics in various aspects. These differences result in interesting biological and clinical consequences.

While mtDNA mutations are typically inherited from the mother, they can also occur de novo. Moreover, a number of nuclear genes are responsible for proper maintenance of mtDNA and mutations in these genes can therefore lead to quantitative (mtDNA depletion) and qualitative defects (mtDNA deletions) defects in mtDNA [9]. In this review, we will focus on renal diseases caused by genetic mutations in mitochondrial DNA.

## Mitochondrial genome and genetics

### Genome

**Fig. 1:**
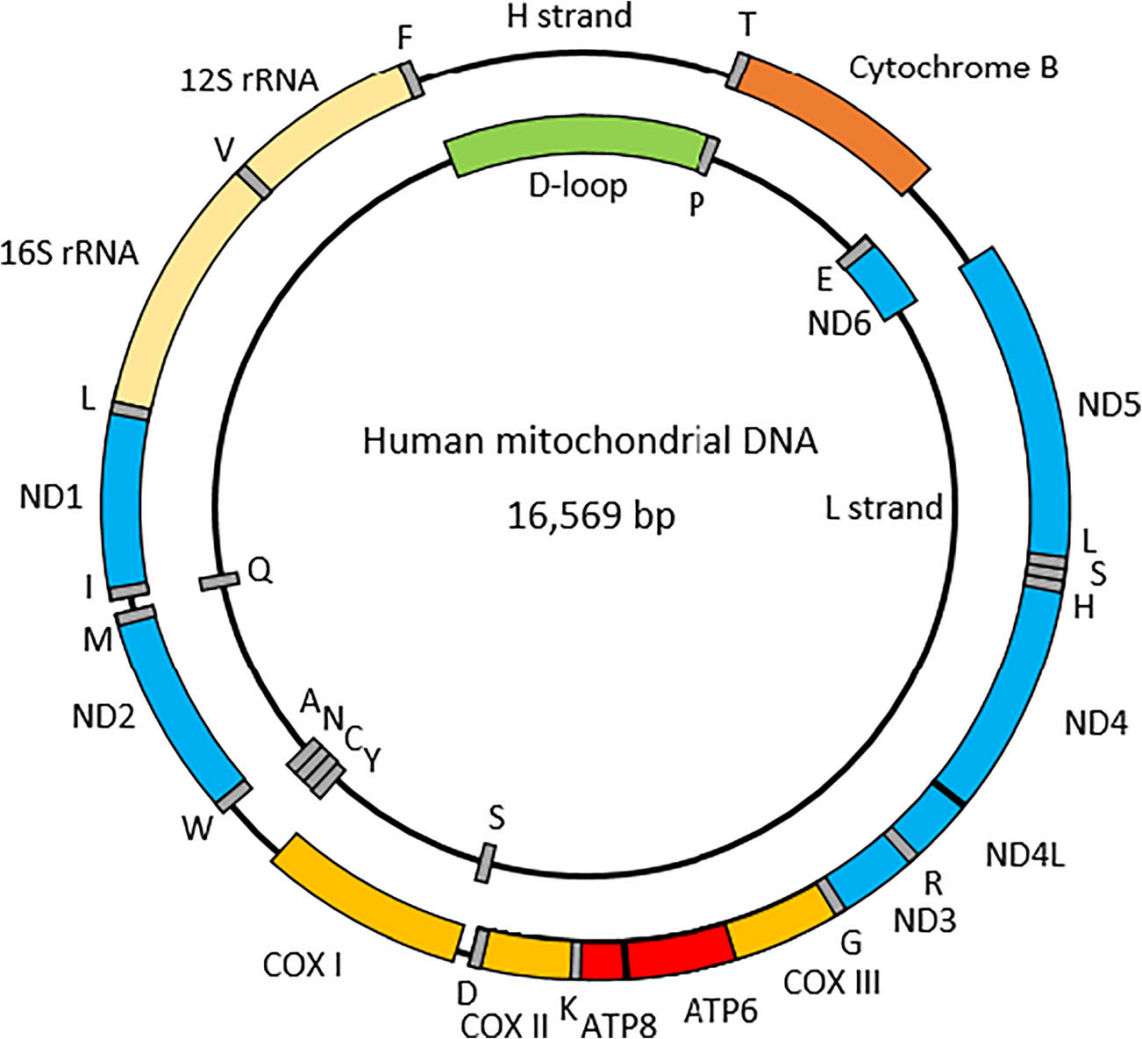
The circular human mitochondrial genome. The letters represent the tRNA genes. ND: NADH dehydrogenase; COX: Cytochrome C Oxidase; ATP6/8: ATP synthase genes 6 and 8

The mitochondrial genome is a circular, double-stranded DNA molecule with a length of 16,569 base pairs. It contains 37 genes, encoding 22 tRNA, 2 rRNAs and 13 polypeptides (Fig. 1) [10]. Unlike the nuclear DNA, there are no intervening sequences and the entire mtDNA is either coding or involved in the regulation of transcription [11]. Both the tRNAs and rRNAs are involved in the intramitochondrial synthesis of proteins. The polypeptides are part of the five complexes involved in oxidative phosphorylation (OXPHOS). The complexes can be divided into two parts, complexes I–IV constitute the electron transport chain, while complex V is involved in the generation of ATP [12]. Approximately 1500 proteins are localised inside the mitochondria, of which 90 are involved in OXPHOS; most are encoded by nuclear genes [13]. Indeed, complex II is completely encoded by nuclear DNA, while the other complexes contain subunits that are derived from both nuclear and mitochondrial DNA (Table 1) [12]. There is also evidence that nuclear tRNAs are transported into mitochondria [19]. This dual genetic control contributes to the heterogeneity in clinical phenotypes.

**Table 1.** The number of nuclear and mitochondrial genes for each respiratory complex

Complex	Genes nuclear DNA	Genes mitochondrial DNA	Total genes
Complex I [14] (Blue)	38	7	45
Complex II [15]	4	0	4
Complex III [16] (Orange)	10	1	11
Complex IV [17] (Yellow)	10	3	13
Complex V [18] (Red)	14	2	16

The first column refers to the colour of the genes in Fig. 1

**Table 2.** Overview of mitochondrial mutations reported in patients with proximal tubular dysfunction

Category	Mutation	Reference
Isolated proximal tubulopathy	2.8 kbp deletion	Szalbocs et al. [55]
KSS	5 kbp deletion	Shoffner et al. [50]
KSS	5.4 kbp deletion	Mori et al. [48]
KSS/PMPS	7.4 kbp deletion	Lee et al. [54]
PMPS	3.3 kbp deletion	Solano et al. [45]
PMPS	4977 bp deletion	Niaudet et al. [51]
PMPS	5.7 kbp deletion	Majander et al. [52]
CCO	7.3 kbp deletion	Au et al. [53]

Transcription of mtDNA occurs continuously on both strands, independent of the cell cycle and it also occurs in non-dividing cells. The strands are called heavy (H), encoding for 12 protein subunits, 2 rRNAs, and 14 tRNAs, and light (L), which encodes for 1 mRNA and 8 tRNAs [20]. The main noncoding region is called the displacement loop (D-loop), which controls addition, replication, transcription and translation of mtDNA [11]. Transcription of mtDNA is dependent on the association between DNA-directed polymerase RNA mitochondrial (POLRMT) and two initiation factors: mitochondrial transcription factor A (TFAM) and mitochondrial transcription factor B1 or B2 (TFB1M or TFB2M) [21]. The expression of the mitochondrial genome is initiated by the transcription of mtDNA from bidirectional heavy- and light-strand promoters to produce polycistronic transcripts [22]. The role of TFAM is to recruit POLRMT to the promoter initiation site in order for TFB2M to melt the promoter [23]. For the elongation stage, POLMRT requires transcription elongation factor (TEFM), which promotes POLRMT to form longer transcripts [24]. MtDNA replication is accomplished by various nuclear-encoded proteins. One of the proteins responsible is DNA polymerase γ (POLγ). POLγ is a heterotrimer with two subunits POLγA, which proofreads newly synthesised DNA, and POLγB, which enhances interactions with the DNA template. Mutations in POLγA lead to the accumulation of genetic alterations in mtDNA [25].

### Genetics

Within a eukaryotic cell, multiple mitochondria exist, that each contains 1 to 15 mtDNA molecules. Mutations that affect all mtDNA copies are termed homoplasmic; mutated and wild-type DNA are also able to coexist in the same cell and are thus referred to as heteroplasmic. In contrast to nuclear DNA, the replication of mtDNA is not linked to the cell cycle, which allows some templates to replicate more than once during each cycle and others not at all [26]. In order for the mitochondria to become dysfunctional, a minimum amount of the mutated mtDNA has to be present in the cell, which is also referred to as the threshold level. This level, however, can be different between tissues due to the divergent energy dependence [27]. The threshold level of mutation is important for the clinical manifestations of the disease.

Mitochondrial DNA is inherited exclusively from maternal egg cells, because the paternal mitochondria and their DNA are actively eliminated [28]. As a result, fathers with mtDNA mutations are at no risk of transmitting the defect to their offspring. In addition, a mutation can occur de novo [29]. The risk of disease in offspring is dependent on the type of mutation and heteroplasmy in the mother. In the case of a homoplasmic mutation, all the maternal offspring will inherit the affected mtDNA. The penetrance, however, can be variable between patients, because it is also dependent on interactions with nuclear DNA. This is consistent with the role of nuclear genetic modifiers. Environmental factors can also contribute to the manifestation of the phenotype. When the mother carries a heteroplasmic mutation, both the normal and mutant mtDNA are randomly distributed to the daughter cells. This can lead to different levels of mutated mtDNA between offspring, depending on the number of mutated copies in the respective oocyte. But, as the mutated copies are randomly distributed during cell division after fertilisation, it can also lead to divergent mutant load between the various tissues in the offspring. This distribution of mutant mitochondria, however, is subject to the “bottleneck effect”: during germ cell development, there is a reduction in mtDNA molecules, so that only a small number are present in oocytes [30]. Depending on the number of mutant mtDNA that was randomly chosen for a given cell, different ratios of healthy and mutant mitochondria will be present in the various oocytes [31]. In addition, there may be focal destruction of mutant mtDNA by autophagy or elimination of cells with a high load of mutant with mitochondria because of their reduced fitness [32]. These factors may explain why mitochondrial mutations do not affect all offspring, or even organs within the same individual, equally.

The mutation rate in the mitochondrial genome is 5 to 10 times greater than in the nuclear genome [33]. This can be attributed to the high amount of reactive oxygen species (ROS) in the mitochondria, that are produced during the ATP synthesis, which can damage mtDNA. Spontaneous mutations from ROS affect the mitochondrial genome more extensively than the nuclear genome. Reasons for this include the absence of protective histones in the mitochondria and the lack of efficient internal repair mechanisms for DNA in the mitochondrial genome compared to the nuclear genome. These mutations can lead to mitochondrial dysfunction, leading to a further increase in ROS production [34]. This progressive damage in the mitochondrial genome and the consequent decrease in mtDNA copy number is thought to contribute to ageing and has been associated with cardiovascular and chronic kidney disease [35]. Generally, the mutations in mtDNA are mostly located in the genes involved in maintenance, transcription, and translation of mtDNA such as transfer and ribosomal RNAs as opposed to in the genes encoding for the OXPHOS subunits [36]. Yet, regardless of the location of the mtDNA mutation, renal manifestations and especially proximal tubulopathy have been reported.

## Tubular defects

### Proximal tubular dysfunction

A generalised impairment of proximal tubule function is called renal Fanconi syndrome (RFS), and is characeterised by decreased reabsorption of various filtered solutes, such as electrolytes, sugars, amino acids and proteins [37]. Features typically associated with RFS include low molecular weight proteinuria, generalised aminoaciduria, hypophosphatemia (with or without bone disease), non-diabetic glycosuria, hypouricosuria and proximal renal tubular acidosis. If a renal biopsy is performed it will typically show dysmorphic mitochondria on electron microscopy [38]. However, the manifestation of RFS can differ between these patients, depending on the severity of the tubular dysfunction. Accordingly, the renal manifestations could be limited to a subset of the beforementioned abnormalities [37]. Besides mitochondrial mutations, this disorder can result from many other causes such as inherited metabolic disease or toxic agents.

### Mutations

Renal manifestations without any extra-renal dysfunction may be the first clinical symptom of mitochondrial disorders, since proximal tubule cells are highly dependent on ATP provision [39]. However, ultimately, multiple organs may be affected with consequent neurological symptoms, myopathy, deafness or cardiac problems [40–43]. In fact, these other symptoms may predominate and kidney involvement is only recognised later: multiple cases have been reported of patients initially diagnosed with Pearson’s marrow-pancreas syndrome (PMPS), defined by sideroblastic anaemia and pancreas dysfunction, who also developed RFS [44–46]. Similar reports exist for Kearns-Sayre (KSS) [47–50] and Leigh syndrome [40]. Several studies have found large mtDNA deletions in mitochondrial DNA to be the underlying cause, ranging from 2.7 to 7.4 kbp deletions [48, 51–55]. Overall, these patients show phenotypic similarities to Pearson and Kearns-Sayre syndromes. Furthermore, RFS can be a symptom of cytochrome C oxidase (CCO) deficiency [41, 56]. After analysis of the respiratory chain complexes using muscle biopsies, complex III and IV were found to have a decreased activity [39, 42, 43, 48].

### Distal tubular dysfunction

In addition to proximal tubulopathy, there have also been patients described suffering from specific electrolyte disturbances, most commonly hypomagnesemia and hypokalaemia. The key nephron segment for regulated magnesium reabsorption is the distal convoluted tubule (DCT) and its high-energy consumption makes it vulnerable to mitochondrial dysfunction [57]. Several cases have been described of patients suffering from electrolyte disturbances, consistent with DCT dysfunction. Again, many of these patients have multisystem disorders, such as Kearns-Sayre syndrome [58–60] or Leigh syndrome [61].

### Mutations

Numerous patients were found to have hypoparathyroidism leading to hypocalcaemia and hyperphosphatemia [58, 60, 62–68]. Magnesium is an important co-factor for the release of PTH hormone and thus, hypoparathyroidism is a common consequence of hypomagnesemia [69]. Magnesium levels were, however, not checked in every patient with hypoparathyroidism, but were significantly decreased in multiple cases [58, 61, 62, 66, 67]. Furthermore, most patients with hypomagnesemia also suffered from hypokalaemia [61, 62, 67]. Overall, these patients also suffered from sensorineural hearing deficits and myopathic symptoms. In a few cases, a clear deletion in mitochondrial DNA was found, ranging from 6 kbp to 8.8kbp (Table 3) [49, 65, 67, 70, 71].

**Table 3.** Overview of mitochondrial mutations reported in patients with distal tubular dysfunction.

Category	Mutation	Reference
Hypomagnesemia and hypokalaemia	T4291C	Wilson et al. [8]
Hypomagnesemia and hypokalaemia	8.8 kbp deletion	Goto et al. [49]
Hypoparathyroidism	7813 bp deletion 8348 bp deletion 8587 bp deletion 9485 bp deletion	Wilichowski et al. [65]
Hypoparathyroidism	6 kbp deletion	Lee et al. [67]
Hypoparathyroidism	6741 bp deletion	Isotani et al. [68]
Hypoparathyroidism with hypomagnesemia and hypokalaemia	8661 bp deletion	Emma et al. [70]
Tubulopathy with PMPS	8034 bp deletion	Van Ouweland et al. [71]

**Table 4.** Overview of mitochondrial mutations reported in patients with nephrotic syndrome

Category	Mutation	Reference
FSGS	A3243G	Dinour et al. [73]
End stage renal disease (FSGS)	A3243G	Mima et al. [74]
FSGS	A4269G	Taniike et al. [76]
FSGS	G5538A	Lim et al. [77]
FSGS	A5728G	Meulemans et al. [79]
FSGS	A5843G	Scaglia et al. [78]
FSGS	2905 bp deletion	Becher et al. [80]

A classical description of a mitochondrial cytopathy with DCT dysfunction was described in a large pedigree [8]. Symptoms included hypertension, hypercholesterolemia and hypomagnesemia with hypokalaemia and hypocalciuria, the electrolyte abnormalities being typical for DCT dysfunction. The symptoms segregated with a mtDNA mutation T4291C (Table 3) located 5' to the anticodon in the mitochondrial tRNA^Ile^ gene. Further symptoms included migraine headache, sensorineural hearing loss and hypertrophic cardiomyopathy, which are all phenotypes associated with mitochondrial dysfunction.

**Table 5.** Overview of mitochondrial mutations reported in patients with tubulointerstitial nephritis

Category	Mutation	Reference
TIN	A547T	Connor et al. [85]
TIN	G586A	D’Aco et al. [83]
TIN	A608G	Tzen et al. [84]
TIN	T616C	Connor et al. [85]
TIN	A5656G	Zsurka et al. [86]

## Non-tubular manifestations

### Nephrotic syndrome

It is not only tubular function that is affected by mitochondrial cytopathies in the kidney. Podocytes also have mitochondria and are energy-dependent. Steroid-resistant NS is a common manifestation of mitochondrial dysfunction associated with coenzyme Q deficiency, which is important to recognise, as it is treatable by ubiquinone supplementation [72]. But nephrotic syndrome (NS) can also be seen with mtDNA mutations. Focal segmental glomerulosclerosis (FSGS) is a typical histological feature seen in degenerative glomerular disorders.

### Mutations

The point mutation m.A3243G, affecting tRNA leucine, is known in patients suffering from myopathy, encephalopathy, lactic acidosis, and stroke-like episodes (MELAS). This mutation has also been described in patients with FSGS [73]. Interestingly, a patient suffering from MELAS with the A3234G mutation was found to have end-stage renal disease with glomerulosclerosis and interstitial fibrosis [74]. This mutation is of particular interest in adults with mtDNA-related disease, as it is the most commonly found mutation [75]. Patients typically present with diabetes and/or hearing loss and the spectrum of renal manifestation besides nephrotic syndrome can include also proximal and/or distal impairment [75]. Different point tRNA mutations can also result in FSGS [76–79]. Besides point mutations, a 2,905 bp deletion was found to result in FSGS, followed by necrotising nephritis with chronic interstitial nephritis [80].

### Tubulointerstitial nephritis

Tubulointerstitial nephritis (TIN) is characterised by the infiltration of the kidney interstitium by inflammatory cells, which can ultimately cause reduced excretory renal function [81]. The phenotype is generally kidney failure and low molecular weight (‘tubular’) proteinuria, which are signs of proximal tubular dysfunction [82]. It is most often caused by drug reactions; however, it may also be caused by infections or systemic disease. Dysregulation of apoptosis has been proposed as a mechanism of pathogenesis of inflammatory processes in mitochondrial TIN. Interestingly, a mitochondrial mutation in the renal epithelial tissue may lead to inflammation while the liver, heart and brain remain unaffected.

### Mutations

In patients with tubulointerstitial nephritis, multiple mutations in tRNA^Phe^ were identified [83–85]. Moreover, in a family diagnosed with severe progressive tubulointerstitial nephritis the mutation A5656G was detected [86].

## Genotype-phenotype

When reviewing mutations in the three clinical categories, it becomes apparent that there is no obvious genotype-phenotype correlation: large mitochondrial deletions can be associated with proximal and distal tubular dysfunction, as well as nephrotic syndrome. Similarly, the most common mitochondrial mutation, A3234G, has initially been associated primarily with FSGS, but a more comprehensive study of adults with mitochondrial disease showed manifestations also included hypophosphatemia, elevated urinary retinol-binding protein and hypomagnesemia, as well as no obvious renal involvement [75]. This variability was also reflected in the extra-renal manifestations, which ranged from maternally inherited diabetes and deafness to MELAS and myoclonic encephalopathy with ragged red fibres.

## Treatment

Since there is not yet a cure for mitochondrial diseases, the current approach is to treat these patients with the aim to alleviate symptoms and slow progression of the disease. Typically, treatment consists of dietary supplements, predominantly antioxidants, such as vitamin C, E, and K (40–160 mg/day), because they are thought to be effective against the damage caused by ROS generation [87]. These vitamins are electron acceptors, which allows them to bypass complex III deficiencies. However, there is no sufficient evidence for the actual benefit [88]. Riboflavin (50–400 mg/day), also known as vitamin B_2_, acts as a flavoprotein precursor in complexes I and II and is shown to be efficient in the C-1 and C-II deficiencies [89]. Folic acid also belongs to the B vitamins family and is involved in protein synthesis in mitochondria. Folate deficiency is common in patients with mitochondrial cytopathies and is often found in patients with Kearns-Sayre syndrome [90]. Furthermore, Coenzyme Q10 (CoQ10) (80–300 mg/day) is most often prescribed for patients with complex III deficiencies, but also affects complexes I and II. It is important for the mitochondrial electron transport chain, because it can move electrons from complex I to II and transfer them to complex III [91]. Other treatments are α-lipoic acid (suggested dosage 5–200 mg/day), which is an antioxidant that can decrease the ROS generated by OXPHOS, therefore decreasing oxidative stress in mitochondria [92], and L-Arginine (150–300 mg/kg/day), a nitric oxide precursor [93, 94]. While these treatments focus on improving the function of the mitochondria, organ-specific interventions, such as kidney transplantation, can also be used as treatment. Treating mitochondrial diseases, however, remains challenging, because the phenotype of the disease in each family is quite variable, and so are the responses to medications. Nuclear genetic modifiers could influence the response drastically.

Patients with mitochondrial disorders can receive genetic counselling to try to prevent disease transmission. However, due to the complexity of the inheritance of mtDNA this remains difficult. To avoid transmission, the most reliable method is to use a donor oocyte. The major limitation is the lack of a child who is biologically related to both parents. Another approach is pronuclear transfer. This technique involves the transfer of the pronuclei from one zygote to another, which results in a zygote containing nuclear DNA from the parents but mtDNA from a donor [95].

## Conclusion and future perspective

In summary, the identification of the different mutations in mitochondrial DNA has enabled us to have a better understanding of the effect of mitochondrial dysfunction on target tissues, including the renal epithelium. A proximal tubulopathy is the most common tubular manifestation, but other patterns of electrolyte abnormalities have also been reported suggesting the involvement of other tubular segments, especially the DCT. Moreover, non-tubular defects due to mitochondrial mutations have been described. A better understanding of the precise disease mechanisms is needed to facilitate improved treatments.
